# Supplementary material for: Operability-economics trade-offs in adsorption-based CO2 capture processes
Source: Commun Eng. 2024 Jul 5;3:94. doi: 10.1038/s44172-024-00244-x (PMC11226646; doi:10.1038/s44172-024-00244-x)
Supplement: Supplementary file 1 — Supplementary Material [file 44172_2024_244_MOESM1_ESM.pdf]

Supplementary information for "Operability-economics trade-offs in adsorption-based CO_2 capture processes"

Steven Sachio, Adam Ward, Ronny Pini,* and Maria M. Papathanasiou*

Department of Chemical Engineering, Imperial College London, South Kensington, SW7 2AZ, UK

E-mail: r.pini@imperial.ac.uk; maria.papathanasiou11@imperial.ac.uk

In the Supplementary Note 1, we provide the material and energy balance equations comprising the 1D adsorption column model. In the Supplementary Note 2, we provide the definitions of all non-dimensional variables and groups used in the adsorption column model. In the Supplementary Note 3, we provide the boundary conditions used to solve the adsorption column model which represent the 4-step pressure-vacuum swing adsorption cycle. In the Supplementary Note 4, we provide details of the calculations of all process key performance indicators. In the Supplementary Note 5, we provide the isotherm model and parametrisation for the description of CO_2/N_2 adsorption equilibrium on zeolite 13X adsorbent. In the Supplementary Note 6, we provide the simulation parameters for the adsorption process cycle. In the Supplementary Note 7, we provide the specification of the 1,000 MW coal fired power plant and the supporting basis calculations. In the Supplementary Note 8, we provide the input parameters to the economic assessment. In the Supplementary Note 9, we provide full details of the process scale-up and economic assessment procedure. In the Supplementary Note 10, we provide the parametric bounds used for carrying out process

optimisation. In the Supplementary Note 11, we provide the process scale-up results for the two process designs proposed in the main text.

Supplementary Note 1

Material & energy balance equations

Table S1: The system of non-dimensional partial differential equations representing the material and energy balances of the 1D adsorption dynamics model.

Overall material balance:	$\frac{\partial \bar{p}}{\partial \tau} - \frac{\bar{p}}{\bar{T}}\frac{\partial \bar{T}}{\partial \tau} = -\bar{T}\frac{\partial}{\partial Z}\left(\frac{\bar{p}\bar{v}}{\bar{T}}\right) - \psi\bar{T}\sum_{i=1}^{n_\mathrm{c}}\frac{\partial x_i}{\partial \tau}$
Component material balance:	$\frac{\partial y_i}{\partial \tau} + \frac{y_i}{\bar{p}}\frac{\partial \bar{p}}{\partial \tau} - \frac{y_i}{\bar{T}}\frac{\partial \bar{T}}{\partial \tau} = \frac{1}{\mathrm{Pe}}\frac{\bar{T}}{\bar{p}}\frac{\partial}{\partial Z}\left(\frac{\bar{p}}{\bar{T}}\frac{\partial y_i}{\partial Z}\right) - \frac{\bar{T}}{\bar{p}}\frac{\partial}{\partial Z}\left(\frac{y_i\bar{p}\bar{v}}{\bar{T}}\right) - \frac{\bar{T}}{\bar{p}}\psi\frac{\partial x_i}{\partial \tau}$
Solid-phase material balance:	$\frac{\partial x_i}{\partial \tau} = \alpha_i(x_i^* - x_i)$
Pressure drop:	$-\frac{\partial \bar{p}}{\partial Z} = \frac{150}{4r_\mathrm{p}^2}\left(\frac{1-\epsilon}{\epsilon}\right)^2\frac{v_0 L}{p_0}\mu\bar{v}$
Column energy balance:	$\frac{\partial \bar{T}}{\partial \tau} + \Omega_2\frac{\partial \bar{p}}{\partial \tau} = \Omega_1\frac{\partial^2 \bar{T}}{\partial Z^2} - \Omega_2\frac{\partial}{\partial Z}\left(\bar{p}\bar{v}\right) + \sum_{i=1}^{n_\mathrm{c}}\left[\left(\sigma_i - \Omega_3\bar{T}\right)\frac{\partial x_i}{\partial \tau}\right] - \Omega_4\left(\bar{T} - \bar{T}_\mathrm{w}\right)$
Wall energy balance:	$\frac{\partial \bar{T}_\mathrm{w}}{\partial \tau} = \Pi_1\frac{\partial^2 \bar{T}_\mathrm{w}}{\partial Z^2} + \Pi_2\left(\bar{T} - \bar{T}_\mathrm{w}\right) - \Pi_3\left(\bar{T}_\mathrm{w} - \bar{T}_\mathrm{a}\right)$

Supplementary Note 2

Non-dimensionalisation and dimensionless groups

The non-dimensional variables of the adsorption dynamics model are defined as:

$$\bar{p} = \frac{p}{p_0}, \bar{T} = \frac{T}{T_0}, \bar{T}_{\mathrm{w}} = \frac{T_{\mathrm{w}}}{T_0}, \bar{T}_{\mathrm{a}} = \frac{T_{\mathrm{a}}}{T_0}, x_i = \frac{q_i}{q_{\mathrm{s},0}}$$

$$\bar{v} = \frac{v}{v_0}, Z = \frac{z}{L}, \tau = t\frac{v_0}{L}$$

$$p_0 = p_{\mathrm{F}}, T_0 = T_{\mathrm{F}}, v_0 = v_{\mathrm{F}}, q_{\mathrm{s},0} = q_{\mathrm{sb,N_2}}$$

Note that the pressure values used in the model are input as absolute pressures, unless indicated otherwise. The dimensionless groups of the adsorption dynamics model are defined as:

$$\psi = \frac{RT_0\rho_{\mathrm{b}}q_{\mathrm{s},0}}{p_0\epsilon} \tag{1}$$

$$Pe = \frac{v_0 L}{D_{\mathrm{L}}} \tag{2}$$

$$\alpha_i = \frac{k_i L}{v_0} \tag{3}$$

$$\Omega_1 = \frac{\left(\frac{K_{\mathrm{z}}}{v_0 L}\right)}{\rho_{\mathrm{b}}\left(C_{\mathrm{p,s}} + C_{\mathrm{p,a}}q_{\mathrm{s},0}\sum_{i=1}^{n_{\mathrm{c}}} x_i\right)} \tag{4}$$

$$\Omega_2 = \frac{\left(\frac{C_{\mathrm{p,g}}p_0}{RT_0}\right)}{\frac{\rho_{\mathrm{b}}}{\epsilon}\left(C_{\mathrm{p,s}} + C_{\mathrm{p,a}}q_{\mathrm{s},0}\sum_{i=1}^{n_{\mathrm{c}}} x_i\right)} \tag{5}$$

$$\Omega_3 = \frac{C_{\mathrm{p,a}}q_{\mathrm{s},0}}{\left(C_{\mathrm{p,s}} + C_{\mathrm{p,a}}q_{\mathrm{s},0}\sum_{i=1}^{n_{\mathrm{c}}} x_i\right)} \tag{6}$$

$$\Omega_4 = \frac{\left(\frac{2h_{\mathrm{in}}L}{r_{\mathrm{in}}v_0}\right)}{\rho_{\mathrm{b}}\left(C_{\mathrm{p,s}} + C_{\mathrm{p,a}}q_{\mathrm{s,0}}\sum_{i=1}^{n_{\mathrm{c}}} x_i\right)} \tag{7}$$

$$\sigma_i = \frac{\left[\frac{q_{\mathrm{s,0}}}{T_0}\left(-\Delta U_{\mathrm{b},i} + RT_0\bar{T}\right)\right]}{C_{\mathrm{p,s}} + C_{\mathrm{p,a}}q_{\mathrm{s,0}}\sum_{i=1}^{n_{\mathrm{c}}} x_i} \tag{8}$$

$$\Pi_1 = \frac{K_{\mathrm{w}}}{\rho_{\mathrm{w}}C_{\mathrm{p,w}}v_0L} \tag{9}$$

$$\Pi_2 = \frac{2r_{\mathrm{in}}h_{\mathrm{in}}}{r_{\mathrm{out}}^2 - r_{\mathrm{in}}^2} \cdot \frac{L}{\rho_{\mathrm{w}}c_{\mathrm{p,w}}v_0} \tag{10}$$

$$\Pi_3 = \frac{2r_{\mathrm{out}}h_{\mathrm{out}}}{r_{\mathrm{out}}^2 - r_{\mathrm{in}}^2} \cdot \frac{L}{\rho_{\mathrm{w}}c_{\mathrm{p,w}}v_0} \tag{11}$$

Supplementary Note 3

We have modelled a typical four step pressure-vacuum swing adsorption (PVSA) cycle for post-combustion carbon capture. A schematic of the operation of the cycle can be seen in Figure S1. The cycle comprises of the following steps; (1) adsorption, (2) forward blowdown, (3) reverse evacuation and (4) feed pressurisation. During the adsorption step, a steady flow of feed gas is established over the bed with a velocity v_{F} at a pressure p_{H}. Throughout this step the bed preferentially adsorbs CO_2 from the feed gas and expels an N_2 rich light product from the product end. In the forward blowdown step, the feed end of the column is closed and the pressure is reduced from p_{H} to an intermediate pressure, p_{I}. During this step, an N_2 rich raffinate is collected from the product end of the column. During the evacuation step, the product end is closed and the pressure of the column is reduced from p_{I} to a low pressure, p_{L}, by a vacuum pump at the feed end of the column. In this step, a high-purity CO_2 product is collected from the feed end. Finally, during the feed pressurisation step, the pressure is raised from p_{L} to p_{H} by adding flue gas to the column from the feed end, with the product end closed. From this point the cycle repeats, carrying out the same sequence of steps until a cyclic steady state (CSS) is achieved.

Adsorption step: boundary conditions

During the adsorption step, both the feed end and product end are open. The velocity at the feed end is the feed velocity, v_{F}:

$$\bar{v}|_{Z=0} = 1 \tag{12}$$

The pressure at the product end is taken to be the high operating pressure, p_{H}:

$$\bar{p}|_{Z=1} = 1 \tag{13}$$

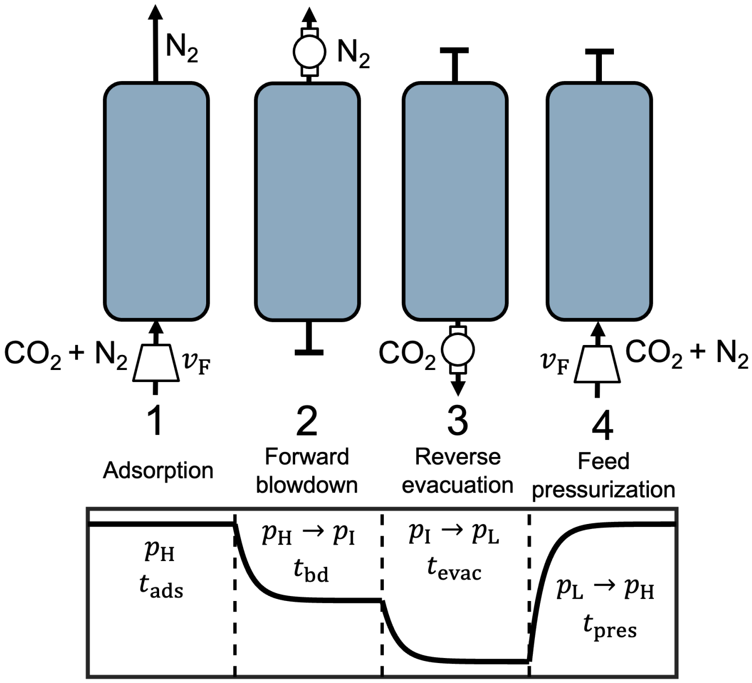

Figure S1: A schematic representation of the four-step PVSA cycle. The steps are (1) adsorption, (2) forward blowdown, (3) reverse evacuation and (4) feed pressurisation.

Each component material balance is subject to the Danckwert's conditions at the feed/product ends of the column. These conditions describe the onset and termination of mechanical dispersion of the gas-phase upon entering/exiting the packed bed. The conditions are given as:

$$\left.\frac{\partial y_i}{\partial Z}\right|_{Z=0} = -\bar{v}|_{Z=0}\mathrm{Pe}\left(y_{i,\mathrm{F}} - y|_{Z=0}\right) \tag{14}$$

$$\left.\frac{\partial y_i}{\partial Z}\right|_{Z=1} = 0 \tag{15}$$

Similarly, using the analogy between diffusive mass transfer and conductive heat transfer, we write the conditions for the energy balance at the feed/product ends as:

$$\left.\frac{\partial \bar{T}}{\partial Z}\right|_{Z=0} = -\bar{v}|_{Z=0}\mathrm{Pe_h}\left(\bar{T}_\mathrm{F} - \bar{T}|_{Z=0}\right) \tag{16}$$

$$\left.\frac{\partial \bar{T}}{\partial Z}\right|_{Z=1} = 0 \tag{17}$$

The wall energy balance is subject to a boundary condition of tight thermal contact between the wall and the ambient surroundings:

$$\bar{T}_{\mathrm{w}}|_{Z=0} = \bar{T}_{\mathrm{w}}|_{Z=1} = \bar{T}_{\mathrm{a}} \tag{18}$$

The conditions applied to the wall energy balance are the same in each step of the cycle.

Forward blowdown step: boundary conditions

During the forward blowdown step, the feed end is closed and the product end is open. Therefore at the feed end we specify:

$$\left.\frac{\partial \bar{p}}{\partial Z}\right|_{Z=0} = 0 \tag{19}$$

$$\left.\frac{\partial y_i}{\partial Z}\right|_{Z=0} = 0 \tag{20}$$

$$\left.\frac{\partial \bar{T}}{\partial Z}\right|_{Z=0} = 0 \tag{21}$$

The conditions applied to the component mass balance and the gas-phase energy balance at the product end are:

$$\left.\frac{\partial y_i}{\partial Z}\right|_{Z=1} = 0 \tag{22}$$

$$\left.\frac{\partial \bar{T}}{\partial Z}\right|_{Z=1} = 0 \tag{23}$$

The pressure profile at the product end during blowdown is specified as the following

exponentially decreasing function:

$$\bar{p}|_{Z=1} = \frac{1}{p_{\mathrm{H}}} \left(p_{\mathrm{I}} + (p_{\mathrm{H}} - p_{\mathrm{I}}) \exp \left[\frac{-\lambda t L}{v_0} \right] \right) \tag{24}$$

Reverse evacuation step: boundary conditions

During the reverse evacuation step, the feed end is open and the product end is closed. Therefore, at the product end we specify:

$$\left. \frac{\partial \bar{p}}{\partial Z} \right|_{Z=1} = 0 \tag{25}$$

$$\left. \frac{\partial y_i}{\partial Z} \right|_{Z=1} = 0 \tag{26}$$

$$\left. \frac{\partial \bar{T}}{\partial Z} \right|_{Z=1} = 0 \tag{27}$$

The conditions applied to the component mass balance and the gas-phase energy balance at the feed end are:

$$\left. \frac{\partial y_i}{\partial Z} \right|_{Z=0} = 0 \tag{28}$$

$$\left. \frac{\partial \bar{T}}{\partial Z} \right|_{Z=0} = 0 \tag{29}$$

The pressure profile at the inlet of the column during blowdown is specified as the following exponentially decreasing function:

$$\bar{p}|_{Z=0} = \frac{1}{p_{\mathrm{H}}} \left(p_{\mathrm{L}} + (p_{\mathrm{I}} - p_{\mathrm{L}}) \exp \left[\frac{-\lambda t L}{v_0} \right] \right) \tag{30}$$

Feed pressurisation step: boundary conditions

During the feed pressurisation step, the feed end is open and the product end is closed. Here, we apply the Danckwert's conditions to the inlet/outlet of the column for the component mass balance and gas-phase energy balance as:

$$\left.\frac{\partial y_i}{\partial Z}\right|_{Z=0} = -\bar{v}|_{Z=0} Pe \left(y_{i,\mathrm{F}} - y|_{Z=0}\right) \tag{31}$$

$$\left.\frac{\partial y_i}{\partial Z}\right|_{Z=1} = 0 \tag{32}$$

$$\left.\frac{\partial \bar{T}}{\partial Z}\right|_{Z=0} = -\bar{v}|_{Z=0} Pe_{\mathrm{h}} \left(\bar{T}_{\mathrm{F}} - \bar{T}|_{Z=0}\right) \tag{33}$$

$$\left.\frac{\partial \bar{T}}{\partial Z}\right|_{Z=1} = 0 \tag{34}$$

Since the product end is closed, the pressure at the product end is given by:

$$\left.\frac{\partial \bar{p}}{\partial Z}\right|_{Z=1} = 0 \tag{35}$$

The pressure profile at the feed end during pressurisation is specified as the following exponentially increasing function:

$$\bar{p}|_{Z=0} = \frac{1}{p_{\mathrm{H}}} \left(p_{\mathrm{H}} - (p_{\mathrm{H}} - p_{\mathrm{L}}) \exp\left[\frac{-\lambda t L}{v_0}\right]\right) \tag{36}$$

Supplementary Note 4

Process key performance indicators

The purity and recovery of CO_2 extracted from the flue gas are given by:

$$\mathrm{Pu}_{\mathrm{CO_2}}\,(\%) = 100 \times \frac{n^{\mathrm{evac}}_{\mathrm{CO_2,out}}}{n^{\mathrm{evac}}_{\mathrm{CO_2,out}} + n^{\mathrm{evac}}_{\mathrm{N_2,out}}} \tag{37}$$

$$\mathrm{Re}_{\mathrm{CO_2}}\,(\%) = 100 \times \frac{n^{\mathrm{evac}}_{\mathrm{CO_2,out}}}{n^{\mathrm{pres}}_{\mathrm{CO_2,in}} + n^{\mathrm{ads}}_{\mathrm{CO_2,in}}} \tag{38}$$

The productivity of the process is given by:

$$\mathrm{Pr} = \frac{n^{\mathrm{evac}}_{\mathrm{CO_2,out}}}{V_{\mathrm{bed}} \cdot t_{\mathrm{cycle}}} \tag{39}$$

Where $V_{\mathrm{bed}} = \pi r_{\mathrm{in}}^2 L$ is the volume of the adsorption bed and $t_{\mathrm{cycle}} = \sum_i t_i$ is the total duration of the four-step cycle. The amounts of CO_2/N_2 input to and extracted from the column are calculated as:

$$n^{\mathrm{evac}}_{\mathrm{CO_2,out}} = \left[\sum_{j=1}^{N} q_{\mathrm{s},0} x_{\mathrm{CO_2},j} \rho_{\mathrm{b}} \left(\pi r_{\mathrm{in}}^2 \Delta Z\right) + \frac{p_0}{T_0} \frac{y_{\mathrm{CO_2},j} \bar{p}_j}{R \bar{T}_j} \epsilon \left(\pi r_{\mathrm{in}}^2 \Delta Z\right)\right]_{t=t_{\mathrm{bd}}} - \tag{40}$$

$$\left[\sum_{j=1}^{N} q_{\mathrm{s},0} x_{\mathrm{CO_2},j} \rho_{\mathrm{b}} \left(\pi r_{\mathrm{in}}^2 \Delta Z\right) + \frac{p_0}{T_0} \frac{y_{\mathrm{CO_2},j} \bar{p}_j}{R \bar{T}_j} \epsilon \left(\pi r_{\mathrm{in}}^2 \Delta Z\right)\right]_{t=t_{\mathrm{evac}}}$$

$$n^{\mathrm{evac}}_{\mathrm{N_2,out}} = \left[\sum_{j=1}^{N} q_{\mathrm{s},0} x_{\mathrm{N_2},j} \rho_{\mathrm{b}} \left(\pi r_{\mathrm{in}}^2 \Delta Z\right) + \frac{p_0}{T_0} \frac{y_{\mathrm{N_2},j} \bar{p}_j}{R \bar{T}_j} \epsilon \left(\pi r_{\mathrm{in}}^2 \Delta Z\right)\right]_{t=t_{\mathrm{bd}}} - \tag{41}$$

$$\left[\sum_{j=1}^{N} q_{\mathrm{s},0} x_{\mathrm{N_2},j} \rho_{\mathrm{b}} \left(\pi r_{\mathrm{in}}^2 \Delta Z\right) + \frac{p_0}{T_0} \frac{y_{\mathrm{N_2},j} \bar{p}_j}{R \bar{T}_j} \epsilon \left(\pi r_{\mathrm{in}}^2 \Delta Z\right)\right]_{t=t_{\mathrm{evac}}}$$

$$n_{\mathrm{CO_2,in}}^{\mathrm{pres}} = \left[\sum_{j=1}^{N} q_{\mathrm{s},0} x_{\mathrm{CO_2},j} \rho_{\mathrm{b}} \left(\pi r_{\mathrm{in}}^2 \Delta Z\right) + \frac{p_0}{T_0} \frac{y_{\mathrm{CO_2},j} \bar{p}_j}{R \bar{T}_j} \epsilon \left(\pi r_{\mathrm{in}}^2 \Delta Z\right)\right]_{t=t_{\mathrm{pres}}} - \tag{42}$$

$$\left[\sum_{j=1}^{N} q_{\mathrm{s},0} x_{\mathrm{CO_2},j} \rho_{\mathrm{b}} \left(\pi r_{\mathrm{in}}^2 \Delta Z\right) + \frac{p_0}{T_0} \frac{y_{\mathrm{CO_2},j} \bar{p}_j}{R \bar{T}_j} \epsilon \left(\pi r_{\mathrm{in}}^2 \Delta Z\right)\right]_{t=t_{\mathrm{evac}}}$$

$$n_{\mathrm{CO_2,in}}^{\mathrm{ads}} = \left[\sum_{j=1}^{N} q_{\mathrm{s},0} x_{\mathrm{CO_2},j} \rho_{\mathrm{b}} \left(\pi r_{\mathrm{in}}^2 \Delta Z\right) + \frac{p_0}{T_0} \frac{y_{\mathrm{CO_2},j} \bar{p}_j}{R \bar{T}_j} \epsilon \left(\pi r_{\mathrm{in}}^2 \Delta Z\right)\right]_{t=t_{\mathrm{ads}}} - \tag{43}$$

$$\left[\sum_{j=1}^{N} q_{\mathrm{s},0} x_{\mathrm{CO_2},j} \rho_{\mathrm{b}} \left(\pi r_{\mathrm{in}}^2 \Delta Z\right) + \frac{p_0}{T_0} \frac{y_{\mathrm{CO_2},j} \bar{p}_j}{R \bar{T}_j} \epsilon \left(\pi r_{\mathrm{in}}^2 \Delta Z\right)\right]_{t=t_{\mathrm{pres}}} + \frac{p_0 v_0}{R T_0} \epsilon \pi r_{\mathrm{in}}^2 \int_0^{t_{\mathrm{ads}}} \left(\frac{\bar{v} \bar{p} y_{\mathrm{CO_2}}}{\bar{T}}\right)\Big|_{Z=1} dt$$

The total energy usage per tonne of CO_2 captured is given by:

$$E_{\mathrm{T}} = \frac{E_{\mathrm{ads}} + E_{\mathrm{bd}} + E_{\mathrm{evac}} + E_{\mathrm{pres}}}{m_{\mathrm{CO_2,out}}^{\mathrm{evac}}} \tag{44}$$

Where $m_{\mathrm{CO_2,out}}^{\mathrm{evac}} = \omega_{\mathrm{CO_2}} n_{\mathrm{CO_2,out}}^{\mathrm{evac}}$ is the mass of CO_2 extracted during the evacuation step. The energy usage by each cycle step is calculated as follows:

$$E_{\mathrm{ads}} = \epsilon \pi r_{\mathrm{in}}^2 \left(\frac{\gamma}{\gamma - 1}\right) v_0 p_0 \int_0^{t_{\mathrm{ads}}} \left(\frac{\bar{v} \bar{p}}{\eta}\right)\Big|_{Z=0} \left[\left(\frac{p_0 \bar{p}|_{Z=0}}{p_{\mathrm{F}}}\right)^{\left(\frac{\gamma-1}{\gamma}\right)} - 1\right] dt \tag{45}$$

$$E_{\mathrm{bd}} = \begin{cases} E_{\mathrm{bd}} = \epsilon \pi r_{\mathrm{in}}^2 \left(\frac{\gamma}{\gamma-1}\right) v_0 p_0 \int_0^{t_{\mathrm{bd}}} \left(\frac{\bar{v}\bar{p}}{\eta}\right)\Big|_{Z=1} \left[\left(\frac{p_{\mathrm{atm}}}{p_0 \bar{p}|_{Z=1}}\right)^{\left(\frac{\gamma-1}{\gamma}\right)} - 1\right] dt, & \text{if} \quad p_0 \bar{p}|_{Z=1} < p_{\mathrm{atm}} \\ 0, & \text{if} \quad p_0 \bar{p}|_{Z=1} \geq p_{\mathrm{atm}} \end{cases} \tag{46}$$

$$E_{\mathrm{evac}} = \epsilon \pi r_{\mathrm{in}}^2 \left(\frac{\gamma}{\gamma - 1}\right) v_0 p_0 \int_0^{t_{\mathrm{evac}}} \left(\frac{\bar{v} \bar{p}}{\eta}\right)\Big|_{Z=0} \left[\left(\frac{p_{\mathrm{atm}}}{p_0 \bar{p}|_{Z=0}}\right)^{\left(\frac{\gamma-1}{\gamma}\right)} - 1\right] dt \tag{47}$$

$$E_{\text{pres}} = \begin{cases} \epsilon \pi r_{\text{in}}^2 \left(\frac{\gamma}{\gamma-1}\right) v_0 p_0 \int_0^{t_{\text{pres}}} \left(\frac{\bar{v}\bar{p}}{\eta}\right)\Big|_{Z=0} \left[\left(\frac{p_0 \bar{p}|_{Z=0}}{p_{\text{F}}}\right)^{\left(\frac{\gamma-1}{\gamma}\right)} - 1\right] dt, & \text{if} \quad p_0\bar{p}|_{Z=0} > p_{\text{F}} \\ 0, & \text{if} \quad p_0\bar{p}|_{Z=0} \leq p_{\text{F}} \end{cases} \tag{48}$$

The adiabatic efficiency of vacuum pumps in the forward blowdown and reverse evacuation steps is given by the following semi-empirical function:[1]

$$\eta(p) = 0.8 \times \frac{19.55p}{1 + 19.55p} \tag{49}$$

Where the pressure, p, is expressed in bar. There is a consensus in the available literature on this approach for the energy usage calculation of compressors and vacuum pumps.[2–7]

Supplementary Note 5

Adsorption equilibrium

To describe the multi-component adsorption of the CO_2/N_2 mixture on zeolite 13X adsorbent, we have applied the extended dual-site Langmuir (DSL) isotherm model, for which the equilibrium amount adsorbed of a species, i, is given by:

$$q_i^* = \frac{q_{\mathrm{b},i} b_i c_i}{1 + \sum_{j=1}^{n_\mathrm{c}} b_j c_j} + \frac{q_{\mathrm{d},i} d_i c_i}{1 + \sum_{j=1}^{n_\mathrm{c}} d_j c_j} \tag{50}$$

Where $q_{\mathrm{b},i}$ and $q_{\mathrm{d},i}$ are the saturation capacities of species i on site 1 and site 2 of the solid surface, respectively. The parameters b_i and d_i are the adsorption equilibrium constants for each of the adsorption sites, which are expressed as a function of temperature using the van't Hoff equation:

$$b_i = b_{i,0} \exp\left(\frac{-\Delta U_{\mathrm{b},i}}{RT}\right) \tag{51}$$

$$d_i = d_{i,0} \exp\left(\frac{-\Delta U_{\mathrm{d},i}}{RT}\right) \tag{52}$$

Where $\Delta U_{\mathrm{b},i}$ and $\Delta U_{\mathrm{d},i}$ are the molar internal energy change upon adsorption for species i on site 1 and site 2, respectively. The molar concentration of species i in the gas phase is calculated using the ideal gas law:

$$c_i = \frac{y_i p}{RT} \tag{53}$$

Table S2: Parameters of the extended dual-site Langmuir isotherm for describing the multi-component adsorption of CO_2/N_2 on zeolite 13X.[2]

**Material**	**Parameter**	$\mathbf{CO_2}$	$\mathbf{N_2}$
Zeolite 13X	q_b [mol/kg]	3.09	5.84
	q_d [mol/kg]	2.54	-
	b_0 [m^3/mol]	8.65×10^{-7}	2.50×10^{-6}
	d_0 [m^3/mol]	2.63×10^{-8}	-
	ΔU_b [J/mol]	$-36,600$	$-15,800$
	ΔU_d [J/mol]	$-35,700$	-

Supplementary Note 6

Simulation parameters

Table S3: Parameters used to simulate the PVSA process for post-combustion carbon capture using the high fidelity mathematical process model. Simulation parameters are obtained from Haghpanah et al. (2013).[2]

Parameter	Value	Units
**Column dimensions:**		
Column length, L	1	[m]
Column inner radius, r_{in}	0.145	[m]
Column outer radius, r_{out}	0.162	[m]
**Feed properties:**		
Feed pressure, p_{F}	$100,000$	[Pa]
Feed temperature, T_{F}	298.15	[K]
Feed molar composition (CO_2), $y_{1,\mathrm{F}}$	0.15	[-]
**Physical properties:**		
Bed density, ρ_{b}	712	[kg/m^3]
Bed voidage, ϵ	0.37	[-]
Particle voidage, ϵ_{p}	0.35	[-]
Particle radius, r_{p}	0.001	[m]
Particle tortuosity, τ_{p}	3	[-]
Molecular diffusivity, D_{m}	1.5×10^{-5}	[m^2/s]
Thermal conductivity of gas, K_{z}	0.09	[J/m/K/s]
Thermal conductivity of wall, K_{w}	16	[J/m/K/s]
Heat capacity of gas phase, $C_{\mathrm{p,g}}$	30.7	[J/mol/K]
Heat capacity of adsorbed phase, $C_{\mathrm{p,a}}$	30.7	[J/mol/K]
Heat capacity of adsorbent, $C_{\mathrm{p,s}}$	$1,070$	[J/kg/K]
Heat capacity of column wall, $C_{p,w}$	502	[J/kg/K]
Density of wall, ρ_{w}	$7,800$	[kg/m^3]
Dynamic viscosity of gas, μ	1.72×10^{-5}	[kg/m/s]
Overall inside heat transfer coefficient, h_{in}	8.6	[J/m^2/K/s]
Overall outside heat transfer coefficient, h_{out}	2.5	[J/m^2/K/s]
Ratio of ideal gas heat capacities, γ	1.4	[-]
Adiabatic efficiency, η	$\eta = \eta(p)$	[-]
Pressure profile time constant, λ	0.5	[/s]

Table S4: Values of fixed operating conditions for process simulations.

**Parameter**	**Value**	**Units**
Adsorption step duration, t_{ads}	50	[s]
Blowdown step duration, t_{bd}	100	[s]
Evacuation step duration, t_{eval}	100	[s]
Low pressure, p_{L}	0.03	[bar]

Supplementary Note 7

Power plant specification

Table S5: Specifications of the typical 1,000 MW coal-fired power plant considered in this study for the techno-economic analysis of the PVSA process.[8]

Parameter	Value	Units
Effluent composition:	15% CO_2, 85% N_2	mol. %
Generation capacity:	1,000	MW
Thermal efficiency:	0.4	-
LHV of coal:	28,000	kJ/kg
Carbon content of coal:	70	% (DAF)
Thermal power:	2,500	MW
Rate of coal consumption:	89.3	kg/s
Molar flow of carbon in flue gas:	5,208	mol/s

Generation capacity

The largest coal fired plants in the world have capacities of 1000's MW, while the smallest are 100's MW. We therefore took a value of 1,000 MW as being representative of a typical power plant size.

Thermal efficiency

The thermal efficiency is the proportion of the thermal energy released by combustion of coal which is converted into useful electricity after considering all components in the system, such as the boiler, turbines, generator and transformers. The thermal efficiency can vary between 35% to 45% depending on how modern the fleet in a given country is. We therefore took a thermal efficiency of 40% as being representative of a typical power plant.

Coal properties

We used a value for the lower heating value of coal of 28,000 kJ/kg$_{\mathrm{coal}}$ and a carbon content of 70% on a mass basis[8]. Both values correspond to a dry ash free (DAF) basis.

Calculation of CO_2 emissions

The thermal power required is calculated as follows:

$$P_{\mathrm{thermal}} = \frac{P_{\mathrm{elec}}}{\eta_{\mathrm{thermal}}} = \frac{1000\mathrm{MW}}{0.4} = 2500\mathrm{MW} = 2.5 \times 10^{6}\mathrm{kJ/s} \tag{54}$$

The required rate of coal supply is then calculated as:

$$\dot{m}_{\mathrm{coal}} = \frac{P_{\mathrm{thermal}}}{\mathrm{LHV}} = \frac{2.5 \times 10^{6}\mathrm{kJ/s}}{28000\mathrm{kJ/kg_{coal}}} = 89.29\mathrm{kg_{coal}/s} \tag{55}$$

The molar flow of carbon in the flue gas is then given by:

$$\dot{n}_{\mathrm{c}} = \frac{\dot{m}_{\mathrm{coal}}\psi_{\mathrm{c}}}{\omega_{\mathrm{c}}} = \frac{89.29 \times 10^{3}\frac{\mathrm{g_{coal}}}{\mathrm{s}} \times 0.7\frac{\mathrm{g_c}}{\mathrm{g_{coal}}}}{12\frac{\mathrm{g_c}}{\mathrm{mol_c}}} = 5208\mathrm{mol_c/s} \tag{56}$$

Assuming complete combustion of the fuel and no side reactions, the molar flow of CO_2 in the flue gas is equal to the molar flow of C. Therefore, we estimate that the 1,000 MW power plant produces 5208 mol/s of CO_2. This is equivalent to approximately 9 million tonnes of CO_2 per year.

Supplementary Note 8

Economic assessment parameters

Table S6: Economic parameters used as inputs to the techno-economic analysis.[9]

Parameter	Value	Units
Discount rate, d	0.08	[-]
Economic lifetime, t	25	[yr]
Electricity unit cost, C_{elec}	0.06	[$/kWh]
Adsorbent unit cost, C_{ads}	1500	[$/tonne]

Supplementary Note 9

Process scale-up

Because an adsorption column must cyclically alternate between adsorption and regeneration modes, multiple adsorption columns must operate in parallel to provide a unit which can continuously accept the flue gas feed. The minimum basic unit which facilitates continuous feed is called a "train" of adsorption columns. Further, because a very large volumetric flow rate of flue gas is produced by the power plant, a single train will not be able to accept a sufficient amount of feed. Therefore, many trains of columns are arranged in parallel to handle the total amount of flue gas. A schematic representation of this process scheme can be found in Fig. S2. To scale-up the adsorption process, we have followed the approach set out by Subraveti et al.[9] The total number of columns which must be arranged in parallel to form a continuously operating train is given by:

$$N_{\text{col}} = \text{ceiling}\left(\frac{t_{\text{cycle}}}{t_{\text{ads}} + t_{\text{pres}}}\right) \tag{57}$$

The minimum number of these trains which must be arranged in parallel to enable processing of the total volume of flue gas is given by:

$$M = \text{ceiling}\left(\frac{\dot{n}_{\text{CO}_2,\text{emitted}}}{\dot{n}^{\text{ads}}_{\text{CO}_2,\text{in}}}\right) \tag{58}$$

Where M is the number of parallel trains, $\dot{n}_{\text{CO}_2,\text{emitted}}$ is the molar flow rate of CO_2 in the flue gas and $\dot{n}^{\text{ads}}_{\text{CO}_2,\text{in}}$ is the molar flow rate of CO_2 which is accepted by a single column during the adsorption step. Since Eq. 57 implies a column schedule for each train in which a single column is always accepting feed gas, the total molar flow of CO_2 which is processed by a train is equal to the rate at which CO_2 is fed to a single adsorption column.

The scaled-up process also requires compressors and vacuum pumps to facilitate the pressure changes throughout the process cycle. Since each train continuously processes flue

gas in one column at a time, the number of compressors required for the scaled-up process is equal to the number of parallel trains, M. The number of vacuum pumps per train for blowdown and evacuation of the adsorption columns during the regeneration steps is given by:

$$N_{\mathrm{v,bd}} = \mathrm{ceiling}\left(\frac{t_{\mathrm{bd}}}{t_{\mathrm{ads}} + t_{\mathrm{pres}}}\right) \tag{59}$$

$$N_{\mathrm{v,evac}} = \mathrm{ceiling}\left(\frac{t_{\mathrm{evac}}}{t_{\mathrm{ads}} + t_{\mathrm{pres}}}\right) \tag{60}$$

Note that separate vacuum pumps must be used to depressurize the columns in the blowdown and evacuation steps, respectively, to prevent cross-contamination of the high purity CO_2 product in the evacuation step with the low purity waste stream from the blowdown step.

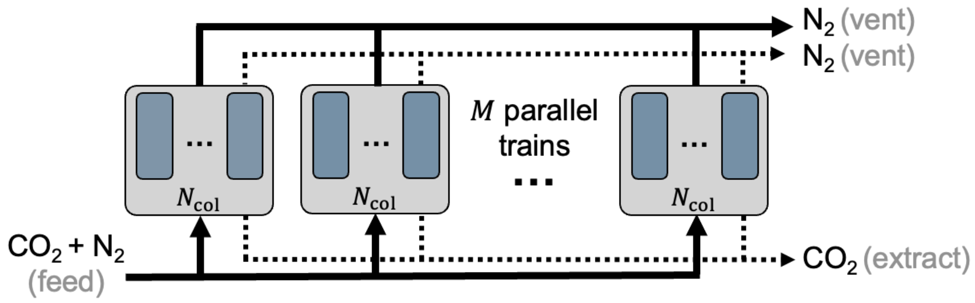

Figure S2: A schematic representation of the scaled-up adsorption process configuration. Adapted from Subraveti et al.[9]

Capital costs

The capital cost of the scaled-up process has been evaluated using a bottom-up approach, as described by Subraveti et al.[9] Firstly, the total direct cost (TDC) is calculated by the sum of the cost of the required equipment. Several additional allowances are then made. A process contingency (15% of TDC) is added to account for potential underestimation of process equipment costs to give the total direct cost including process contingency (TDCPC). We

then include indirect costs (14% of TDCPC) and a project contingency (20% of TDCPC). Indirect costs account for factors such as engineering and consultancy fees. The project contingency is to allow for unforeseen costs and issues which may arise during the construction of the project. This gives the total plant cost (TPC). Finally, an allowance is added for ownership costs (7% of TDCPC) to give the total capital required (TCR). Ownership costs include the costs of buying land, insurance, legal fees and currency exchanges, among several other expenses. The TCR represents the total amount of capital which needs to be raised to build out a project with a given TDC, given the required contingencies. Overall, the allowances made amount to increasing the TDC by approximately 62.15% to give the TCR. We have annualised the TCR using the equivalent annual cost (EAC) approach.[6] The EAC is given by:

$$\mathrm{EAC}\,(\$/\mathrm{yr}) = \frac{\mathrm{TCR}\cdot d}{1-(1+d)^{-t}} \tag{61}$$

Where d is the discount rate and t is the economic lifetime of the project. The most important contributions to the TDC of the scaled-up process are those of the adsorption columns, the compressors, the vacuum pumps and the initial adsorbent.[10] Each of these equipment costs have been estimated from correlations available in the literature.[11]

The base cost of an adsorption column is given by the following correlation:

$$\log_{10}(C_{\mathrm{B,col}}) = 3.4974 + 0.4485\log_{10}(V) + 0.1074\left(\log_{10}(V)\right)^2 \tag{62}$$

Where $V = \pi r_{\mathrm{in}}^2 L$ is the volume of an adsorption column (m^3). The actual column cost is then calculated as:

$$C_{\mathrm{col}}\,(\$) = (2.52 + 1.82 F_{\mathrm{m}} F_{\mathrm{p}}) \times \frac{\mathrm{CEPCI}_{2021}}{\mathrm{CEPCI}_{2001}} \cdot C_{\mathrm{B,col}} \tag{63}$$

Where CEPCI_i is the chemical engineering plant cost index for year i and is used to adjust the base cost of the column for inflation. F_{m} and F_{p} are the material and pressure

factors. For an adsorption column, the material factor is $F_{\mathrm{m}} = 1$. The pressure factor is given by:

$$F_{\mathrm{p}} = \max\left(\frac{\frac{(p_{\mathrm{H}}+1)2r_{\mathrm{in}}}{2(850-1.5(p_{\mathrm{H}}+1))} + 0.00315}{0.0063}, 1.25\right) \tag{64}$$

Where p_{H} is the adsorption pressure (barg) and r_{in} is the column inner radius (m). The base cost of a vacuum pump is given by the following correlation:

$$\log_{10}(C_{\mathrm{B,pump}}) = 3.3892 + 0.0536\log_{10}(\dot{W}) + 0.1536\left(\log_{10}(\dot{W})\right)^2 \tag{65}$$

Where $\dot{W}$ is the maximum shaft power of the vacuum pump (kW). The actual vacuum pump cost is then calculated as:

$$C_{\mathrm{pump}}\,(\$) = 5 \times \frac{\mathrm{CEPCI}_{2021}}{\mathrm{CEPCI}_{2001}} \cdot C_{\mathrm{B,pump}} \tag{66}$$

For the costing of compressors, the cost is broken down into the cost of the compressor itself and the cost of the drive. The base costs of these components are given by the following correlations:

$$\log_{10}(C_{\mathrm{B,comp}}) = 2.2897 + 1.3604\log_{10}(\eta\dot{W}) - 0.1027\left(\log_{10}(\eta\dot{W})\right)^2 \tag{67}$$

$$\log_{10}(C_{\mathrm{B,drive}}) = 1.956 + 1.7142\log_{10}(\dot{W}) - 0.2282\left(\log_{10}(\dot{W})\right)^2 \tag{68}$$

The actual costs of the compressor and drive are then calculated as:

$$C_{\mathrm{comp}}\,(\$) = 2.7 \times \frac{\mathrm{CEPCI}_{2021}}{\mathrm{CEPCI}_{2001}} \cdot C_{\mathrm{B,comp}} \tag{69}$$

$$C_{\mathrm{drive}}\,(\$) = 1.5 \times \frac{\mathrm{CEPCI}_{2021}}{\mathrm{CEPCI}_{2001}} \cdot C_{\mathrm{B,drive}} \tag{70}$$

We can then calculate the TDC by summing the contributions of the costs of the adsorp-

tion columns, vacuum pumps, compressors and the cost of the initial adsorbent:

$$\mathrm{TDC}\,(\$) = N_{\mathrm{col}} M C_{\mathrm{col}} + N_{\mathrm{v,bd}} M C_{\mathrm{pump}}^{\mathrm{bd}} + N_{\mathrm{v,evac}} M C_{\mathrm{pump}}^{\mathrm{evac}} + \\ M(C_{\mathrm{comp}} + C_{\mathrm{drive}}) + N_{\mathrm{col}} M V \rho_{\mathrm{b}} C_{\mathrm{ads}} \tag{71}$$

Operating costs

For the purposes of assessing the relative techno-economic performance of processes utilizing different adsorbent materials, the two most important operating costs to account for are the cost of electricity used to drive the vacuum pumps and compressors, and the cost of regularly replenishing the adsorbent in the system.[10] The cost of electricity is calculated as:

$$\mathrm{OPEX}_{\mathrm{elec}}(\$/\mathrm{yr}) = E_{\mathrm{T}} \mathrm{Re}_{\mathrm{CO_2}} \dot{m}_{\mathrm{CO_2,emitted}} C_{\mathrm{elec}} \tag{72}$$

Where E_{T} is the energy usage of the capture process (kWh/tonne), $\dot{m}_{\mathrm{CO_2,emitted}}$ is the mass flow rate of CO_2 in the flue gas (tonne/yr) and C_{elec} is the unit cost of electricity ($/kWh).

To calculate the cost of replenishing the adsorbent material, it has been assumed that the adsorbent in the system needs to be replaced on an annual basis. This cost can then be taken as a regularly occurring operating expense that we account for once per year. The cost of replacing the adsorbent in the system is calculated as:

$$\mathrm{OPEX}_{\mathrm{ads}}(\$/\mathrm{yr}) = N_{\mathrm{col}} M V \rho_{\mathrm{b}} C_{\mathrm{ads}} \tag{73}$$

Where C_{ads} is the unit cost of the adsorbent material. ZIF-36-FRL belongs to the metal organic framework (MOF) family of adsorbent materials. While there is significant interest in these materials in the academic literature for a variety of applications, most MOFs (including ZIF-36-FRL) are yet to be readily available at a commercial scale. Based on techno-economic evaluations reported in the literature it is estimated that, with economies of scale and mature

demand markets, the unit cost of MOFs could be on the order of tens of thousands of dollars per tonne.[12] The unit cost also depends strongly on the metallic composition of each specifc MOF, which can vary significantly between materials. As a crude approximation, we have optimistically estimated for the purposes of this study that ZIF-36-FRL could cost 10,000 \$/tonne if deployed widely at commercial scale for post-combustion capture.

The total operating cost is then calculated as the sum of the annual electricity and adsorbent replenishment costs:

$$\mathrm{OPEX}\,(\$/\mathrm{yr}) = E_{\mathrm{T}}\mathrm{Re}_{\mathrm{CO_2}}\dot{m}_{\mathrm{CO_2,emitted}}C_{\mathrm{elec}} + N_{\mathrm{col}}MV\rho_{\mathrm{b}}C_{\mathrm{ads}} \tag{74}$$

Therefore, the total annual cost (TAC) of the capture process can be calculated as the sum of the equivalent annual cost of the capital expenditure with the annual operating cost:

$$\mathrm{TAC}\,(\$/\mathrm{yr}) = \mathrm{EAC} + \mathrm{OPEX} \tag{75}$$

The total annual cost may be used in conjunction with the annual amount of CO_2 captured to calculate the cost per tonne of CO_2 captured from the flue gas:

$$C^{\mathrm{cap}}_{\mathrm{CO_2}}\,(\$/\mathrm{tonne}) = \frac{\mathrm{TAC}\,(\$/\mathrm{yr})}{\mathrm{Re}_{\mathrm{CO_2}}\dot{m}_{\mathrm{CO_2,emitted}}(\mathrm{tonne/yr})} \tag{76}$$

Supplementary Note 10

Optimisation bounds

In Table S7, we provide the design decisions and parametric bounds for optimization of the process performance when using the NSGA-II algorithm. A linear inequality constraint is imposed on the optimization to ensure that the high pressure is always larger than the intermediate pressure, to ensure that only physical solution to the process model are obtained:

$$p_{\mathrm{H}} \geq p_{\mathrm{I}} + 1000\,\mathrm{Pa} \tag{77}$$

Table S7: Design decisions and parametric bounds when optimizing process performance using the NSGA-II algorithm.

**Parameter**	p_{H} [bar]	p_{I} [bar]	v_{F} [m/s]
**Lower bound**	1	0.05	0.1
**Upper bound**	10	3	1

Supplementary Note 11

Scale-up of proposed designs

Table S8: Summary of process scale-up for the maximum flexibility design and the relaxed cost optimal design at the respective nominal operating points.

	Maximum flexibility	Cost optimal
Operating conditions		
High pressure, p_H [bar]	6.00	3.55
Intermediate pressure, p_I [bar]	0.765	1.25
Feed velocity, v_F [m/s]	0.794	1.40
Performance indicators		
Purity, $\mathrm{Pu}_{\mathrm{CO}_2}$ [%]	98.3	96.5
Recovery, $\mathrm{Re}_{\mathrm{CO}_2}$ [%]	93.8	90.4
Productivity, Pr [mol/m^3/s]	2.03	1.99
Energy usage, E_T [kWh/tonne]	624	515
Scale-up		
Number of columns per train, N_col [-]	6	6
Number of parallel trains, M [-]	5596	5364
Number of blowdown pumps per train, $N_\mathrm{v,bd}$ [-]	2	2
Number of evacuation pumps per train, $N_\mathrm{v,evac}$ [-]	2	2

References

(1) Maruyama, R. T.; Pai, K. N.; Subraveti, S. G.; Rajendran, A. Improving the performance of vacuum swing adsorption based CO2 capture under reduced recovery requirements. *International Journal of Greenhouse Gas Control* **2020**, *93*.

(2) Haghpanah, R.; Majumder, A.; Nilam, R.; Rajendran, A.; Farooq, S.; Karimi, I. A.; Amanullah, M. Multiobjective Optimization of a Four-Step Adsorption Process for Postcombustion CO2 Capture Via Finite Volume Simulation. *Industrial & Engineering Chemistry Research* **2013**, *52*, 4229–4265.

(3) Subraveti, S. G.; Roussanaly, S.; Anantharaman, R.; Riboldi, L.; Rajendran, A. How much can novel solid sorbents reduce the cost of post-combustion CO_2 capture? A techno-economic investigation on the cost limits of pressure-vacuum swing adsorption. *Applied Energy* **2022**, *306*.

(4) Jiang, H.; Ebner, A. D.; Ritter, J. A. Importance of Incorporating a Vacuum Pump Performance Curve in Dynamic Adsorption Process Simulation. *Industrial & Engineering Chemistry Research* **2020**, *59*, 856–873.

(5) Khurana, M.; Farooq, S. Adsorbent Screening for Postcombustion CO2 Capture: A Method Relating Equilibrium Isotherm Characteristics to an Optimum Vacuum Swing Adsorption Process Performance. *Industrial & Engineering Chemistry Research* **2016**, *55*, 2447–2460.

(6) Zanco, S. E.; Perez-Calvo, J.-F.; Gasos, A.; Cordiano, B.; Becattini, V.; Mozzotti, M. Postcombustion CO2 Capture: A Comparative Techno-Economic Assessment of Three Technologies Using a Solvent, an Adsorbent and a Membrane. *American Chemical Society Engineering Au* **2021**, *1*, 50–72.

(7) Ishibashi, M.; Ota, H.; Akutsu, N.; Umeda, S.; Tajika, M.; Izumi, J.; Yasutake, A.; Kabata, T.; Kageyama, Y. Technology for removing carbon dioxide from power plant flue gas by the physical adsorption method. *Energy Conversion and Management* **1996**, *37*, 929–933, Proceedings of the International Energy Agency Greenhouse Gases: Mitigation Options Conference.

(8) Fu, C.; Anantharaman, R.; Gundersen, T. Thermal efficiency of coal-fired power plants: From theoretical to practical assessments. *Energy Conversion and Management* **2015**, *105*, 530–544.

(9) Subraveti, S. G.; Roussanaly, S.; Anantharaman, R.; Rajendran, A. Techno-economic assessment of optimised vacuum swing adsorption for post-combustion CO2 capture from steam-methane reformer flue gas. *Separation and Purification Technology* **2021**, *256*.

(10) Effendy, S.; Xu, C.; Farooq, S. Optimization of a Pressure Swing Adsorption Process for Nitrogen Rejection from Natural Gas. *Industrial & Engineering Chemistry Research* **2017**, *56*, 5417–5431.

(11) Turton, R.; Shaeiwitz, J. A.; Battacharyya, D.; Whiting, W. B. *Analysis, design and synthesis of chemical processes*; Prentice Hall: Boston, 2018.

(12) Severino, M. I.; Gkaniatsou, E.; Nouar, F.; Pinto, M. L.; Serre, C. MOFs industrialization: a complete assessment of production costs. *Faraday Discussions* **2021**, *231*, 326–341.
